# Severe but reversible impaired diaphragm function in septic mechanically ventilated patients

**DOI:** 10.1186/s13613-022-01005-9

**Published:** 2022-04-11

**Authors:** Marie Lecronier, Boris Jung, Nicolas Molinari, Jérôme Pinot, Thomas Similowski, Samir Jaber, Alexandre Demoule, Martin Dres

**Affiliations:** 1 Médecine Intensive - Réanimation (Département “R3S”), APHP. Sorbonne Université, Hôpital Pitié-Salpêtrière, Paris, France; 2 Neurophysiologie Respiratoire Expérimentale et Clinique, INSERM-UMR S 1158, Sorbonne Université, Paris, France; 3 Département de Médecine Intensive - Réanimation, CHU Montpellier, Montpellier, France; 4 Laboratoire de Physiologie et Médecine Expérimentale du cœur et des Muscles, INSERM U1046-CNRS UMR 9214, Université de Montpellier, Montpellier, France; 5 Department of Medical Information, Hôpital Arnaud de Villeneuve, IMAG U5149, Université de Montpellier, Montpellier, France

**Keywords:** Diaphragm dysfunction, Sepsis, Mechanical ventilation, Sepsis-associated diaphragm dysfunction

## Abstract

**Background:**

Whether sepsis-associated diaphragm dysfunction may improve despite the exposure of mechanical ventilation in critically ill patients is unclear. This study aims at describing the diaphragm function time course of septic and non-septic mechanically ventilated patients.

**Methods:**

Secondary analysis of two prospective observational studies of mechanically ventilated patients in whom diaphragm function was assessed twice: within the 24 h after intubation and when patients were switched to pressure support mode, by measuring the endotracheal pressure in response to bilateral anterior magnetic phrenic nerve stimulation (Ptr,stim). Change in diaphragm function was expressed as the difference between Ptr,stim measured under pressure support mode and Ptr,stim measured within the 24 h after intubation. Sepsis was defined according to the Sepsis-3 international guidelines upon inclusion. In a sub-group of patients, the right hemidiaphragm thickness was measured by ultrasound.

**Results:**

Ninety-two patients were enrolled in the study. Sepsis upon intubation was present in 51 (55%) patients. In septic patients, primary reason for ventilation was acute respiratory failure related to pneumonia (37/51; 73%). In non-septic patients, main reasons for ventilation were acute respiratory failure not related to pneumonia (16/41; 39%), coma (13/41; 32%) and cardiac arrest (6/41; 15%). Ptr,stim within 24 h after intubation was lower in septic patients as compared to non-septic patients: 6.3 (4.9–8.7) cmH_2_O vs. 9.8 (7.0–14.2) cmH_2_O (*p* = 0.004), respectively. The median (interquartile) duration of mechanical ventilation between first and second diaphragm evaluation was 4 (2–6) days in septic patients and 3 (2–4) days in non-septic patients (*p* = 0.073). Between first and second measurements, the change in Ptr,stim was + 19% (− 13–61) in septic patients and − 7% (− 40–12) in non-septic patients (*p* = 0.005). In the sub-group of patients with ultrasound measurements, end-expiratory diaphragm thickness decreased in both, septic and non-septic patients. The 28-day mortality was higher in patients with decrease or no change in diaphragm function.

**Conclusion:**

Septic patients were associated with a more severe but reversible impaired diaphragm function as compared to non-septic patients. Increase in diaphragm function was associated with a better survival.

**Supplementary Information:**

The online version contains supplementary material available at 10.1186/s13613-022-01005-9.

## Background

Because of its potential association with prolonged ventilator dependency and poor clinical outcomes, diaphragm dysfunction is a leading concern in the intensive care unit (ICU) [1–3]. In mechanically ventilated patients, diaphragm dysfunction, defined as a reduction of pressure generating capacity of the diaphragm [4], can occur early after intubation [5] or after several days of mechanical ventilation [1]. This pathophysiological process is commonly termed critical-illness associated diaphragm weakness [6]. The early diaphragm dysfunction that observed on ICU admission is likely caused by the underlying mechanisms leading to ICU, sepsis being the leading cause [5, 7–9]. On the contrary, the later development of diaphragm dysfunction or inability to recover from diaphragm dysfunction can potentially be ascribed to other factors such as ventilator-induced respiratory muscles disuse [10].

The impact of the association between sepsis and mechanical ventilation is controversial. In animal models, sepsis-induced diaphragm dysfunction is alleviated when mechanical ventilation is instituted at the onset of sepsis [11], while it is worsened by concomitant prolonged mechanical ventilation [12, 13]. In humans, there is limited information on the interaction between mechanical ventilation and sepsis. In one cohort, the combination of mechanical ventilation and infection induced diaphragm dysfunction that negatively influenced survival [14]. However, another study reported that diaphragm dysfunction can improve despite persistent exposure to mechanical ventilation [15]. These findings echo the well-described reversible sepsis-induced myocardial dysfunction that occurs septic shock [16].

We hypothesized that, in mechanically ventilated patients, sepsis is associated with a more severe but rapidly reversible reduction in diaphragm pressure generating capacity as compared to non-septic patients. Accordingly, the first objective of the study was to compare the time course of diaphragm function in septic and non-septic patients. In addition, we assessed whether the time course of diaphragm function was associated with the outcome.

## Methods

This is a secondary analysis of two prospective observational studies [5, 17]. The first study was bicentric (Paris, Montpellier) and conducted between December 2008 and July 2009 [5]. The second study was monocentric (Paris) and conducted between November 2014 and June 2015 [17]. The protocols were, respectively, approved by the Comité de Protection des Personnes Ile de France VI and Sud-Méditerrannée II, Montpellier, France (N° 2014-A00715-42 et N° CCPPRB 06 04 03). All patients or their relatives provided written informed consent to participate.

### Patients

Patients older than 18 years were eligible for inclusion in the two studies within the 24 h after oral or nasal endotracheal intubation. Exclusion criteria were contraindications to magnetic stimulation of the phrenic nerves (cardiac pacemaker or implanted defibrillator, chest drain in situ, cervical spine implants); use of neuromuscular blocking agents within the 24 h preceding the first diaphragm assessment (with the exception of succinylcholine used during rapid-sequence induction of anesthesia for intubation); preexisting neuromuscular disorders; cervical spine injury; pregnancy; and a decision to withhold life sustaining treatment.

### Diaphragm function and thickness assessment

In the two studies, first diaphragm function was assessed within 24 h of intubation. Whenever possible these measurements were repeated one or several times until extubation. In the present study, we only included patients in whom (1) two assessments of diaphragm function were available, and (2) the second assessment was performed within range of 24 h after the patients were switched to pressure support mode.

Diaphragm function was measured as the pressure generating capacity of the diaphragm (Ptr,stim) in response to bilateral anterolateral phrenic nerve magnetic stimulation [4, 18, 19]. Two figure-of-eight coils connected to a pair of Magstim® 200 stimulators (Magstim Company, Dyfed, UK) were positioned immediately posterior to the sternomastoid muscles at the level of the cricoid cartilage. Stimulations were delivered at the maximum intensity allowed by the stimulator (100%). Patients were studied in a standardized semi-seated position. Positive end-expiratory pressure (PEEP) was not modified during the measurement. The endotracheal tube was manually occluded at the end of expiration, and the stimulation was performed. The absence of active respiratory efforts during stimulation was confirmed by inspecting the stability of the airway pressure signal. Ptr,stim was defined as the amplitude of the negative pressure wave following stimulation, taken from baseline to peak. It was measured at the external tip of the endotracheal tube, using a linear differential pressure transducer (MP45 ± 100 cm H_2_O, Validyne, Northridge, Calif., USA). The pressure signal was sampled and digitized at 128 Hz (MP30, Biopac Systems, Santa Barbara, Calif., USA or Powerlab, AD Instruments, Bella Vista, Australia) for subsequent data analysis. A Ptr,stim less than 11 cm H_2_O defined diaphragm dysfunction [20, 21].

For the purpose of the present study, we also used ultrasound data collected in patients enrolled in the second study [17]. Diaphragm ultrasound was performed to measure the right hemidiaphragm end-expiratory thickness. The technical description of ultrasound measurements has been reported elsewhere [17]. Briefly, a 5–12 MHz linear array probe was used for all the measurements (Sparq, Philips, Philips Healthcare, Andover, MA, USA). The probe was placed in the right anterior axillary line between the ninth or tenth intercostal space in the sagittal oblique plane. To ensure the best reproducibility between two measurements, the position of the probe was carefully specified by marking the skin. The two-dimensional (2D) mode was initially used to obtain the best approach and identify the diaphragm, which appears as a three-layered structure just superficial to the liver. M-mode was then used to display the motion of the diaphragm, with sweep was set at 10 mm/s. Measurements of end-expiratory thickness were always made on at least three separate breaths visualized on a single M-mode image. The average of all respiratory cycles was retained.

### Data collection

Demographic data (age, sex, body mass index and comorbidities), Sequential Organ Failure Assessment (SOFA) and New Simplified Acute Physiology Score (SAPS 2), primary reason for ventilation, sepsis at inclusion, arterial pressure, heart rate, arterial blood gas, plasma procalcitonin level, microbiological findings, use of vasopressor and ventilator settings were prospectively collected. Total duration of mechanical ventilation, duration of ventilation between measurements, ventilator-free days at 28 days, ICU stay, ICU and 28 days mortality were also recorded.

Sepsis was defined at the time of inclusion according to the Sepsis-3 international guidelines [22], as life-threatening organ dysfunction represented by an increase in the SOFA score of 2 points or more caused by a dysregulated host response to infection. Septic shock was defined when a vasopressor was required to maintain a mean arterial pressure of 65 mmHg or greater and serum lactate level greater than 2 mmol/L (> 18 mg/dL) in the absence of hypovolemia.

### Primary and secondary endpoints

The primary end-point was the change in Ptr,stim between the two measurements (within 24 h after intubation and at the time of switch to pressure support mode). Secondary end points were: the proportion of diaphragm dysfunction at inclusion, change in end-expiratory diaphragm thickness between two diaphragm function measurements and mortality.

### Statistical analysis

Continuous variables are expressed as median (25–75, interquartile range, IQR) and categorical variables are expressed as number and relative frequencies (%). Continuous variables were tested for normality using the Kolmogorov–Smirnov normality test. Due to the retrospective nature of the present analysis, no sample size was deemed necessary.

The study population was first divided into two groups based on the presence of sepsis at inclusion. Patients were also categorized according to the change in Ptr,stim between the two diaphragm function measurements. Increase in diaphragm function was defined as an increase in Ptr,stim > 10% between measurements. Decrease or no change in diaphragm function was defined as an increase in Ptr,stim ≤ 10% or a decrease in Ptr,stim. The 10% change was deemed as clinically relevant.

Clinical characteristics, change in SOFA, change in Ptr,stim, and diaphragm end-expiratory thickness were compared between patients with and without sepsis using Student’s *t* test or Mann–Whitney test for continuous variables depending on distribution and Chi-2 test for categorical variables. Factors associated with “increase in diaphragm function” were identified by univariate analysis. In addition, in order to analyze the level of Ptr,stim as a continuous end-point over time, linear mixed effects models were performed after adjustment on confounding variables and interaction effects. Subject was treated as a random effect. A backward procedure was applied to select the final model. For final comparisons, a two tailed p-value less than or equal to 0.05 was considered statistically significant. All statistical analysis was performed by using Prism 8.4.3 software (GraphPad Software, USA) and R version 3.3.2 (www.R-project.org).

## Results

### Patients characteristics at inclusion

**Fig. 1:**
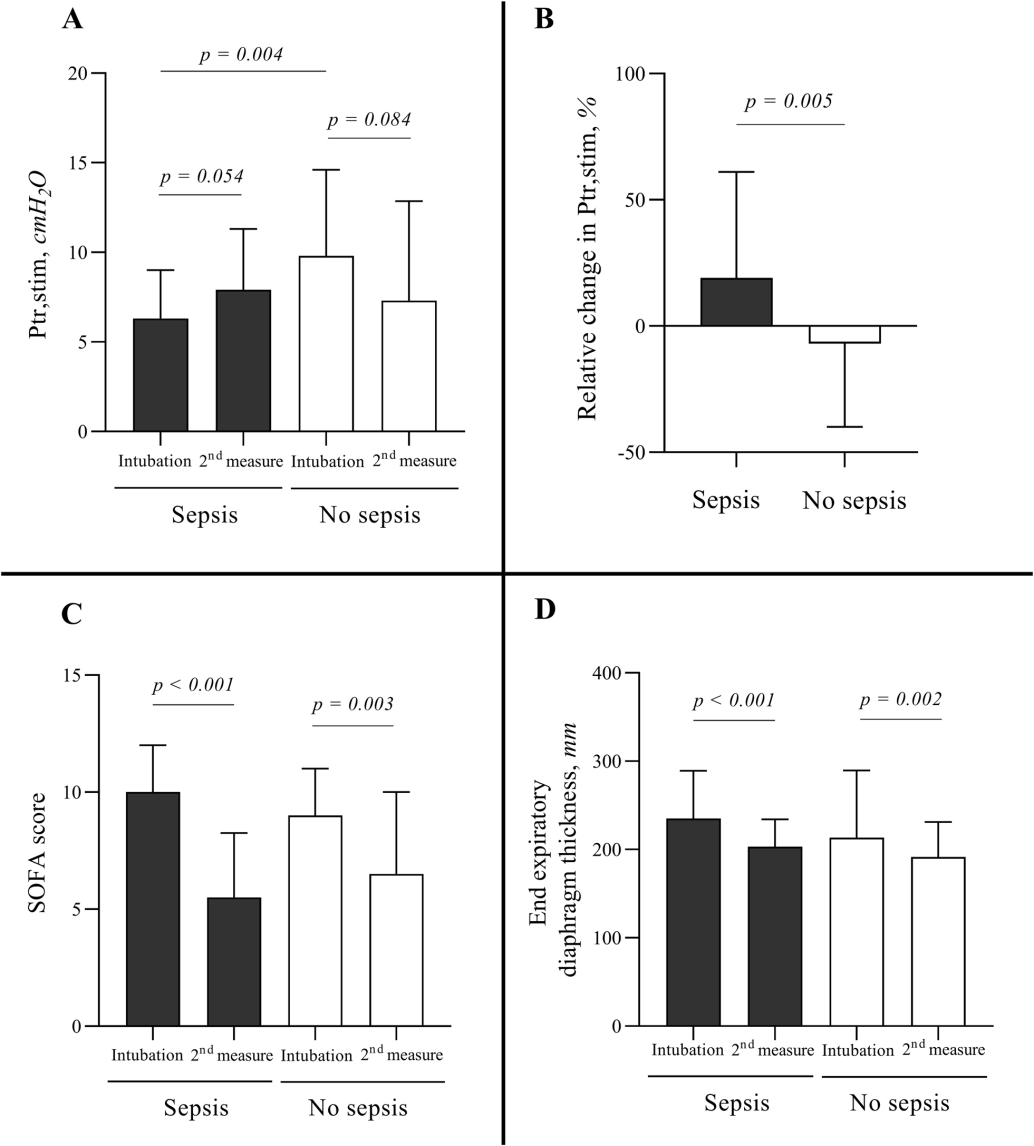
**A** Endotracheal tube pressure induced by bilateral phrenic nerve stimulation during airway occlusion (Ptr,stim) at inclusion (< 24 h of intubation) and at the second measure (pressure support mode) in septic and non-septic patients. *p* identified by Wilcoxon matched-pairs test. **B** Relative changes (%) in endotracheal tube pressure induced by bilateral phrenic nerve stimulation during airway occlusion (Ptr,stim) between the two measurements (inclusion and pressure support mode) in septic and non-septic patients. *p* identified by Mann–Whitney test. **C** SOFA score at inclusion (< 24 h of intubation) and at the second measure (pressure support mode) in septic and non-septic patients. *p* identified by paired t test. **D** End-expiratory diaphragm thickness measured by ultrasound at inclusion (< 24 h of intubation) and at the second measure (pressure support mode) in septic and non-septic patients. *p* identified by Wilcoxon matched-pairs test. Box plot represent median with interquartile range

From the 161 patients included in both cohorts, 92 patients were enrolled in the present study (see Additional file 1: Figure S1). Main characteristics of the patients on inclusion are presented in Table 1. Sepsis was present in 51 (55%) patients. Among them 40/51 (78%) had microbiological evidence of infection (see Additional file 1: Table S1). Lower respiratory tract infection (41/51, 80%) and bloodstream infection (11/51, 22%) were the two most frequent infections. Plasma procalcitonin concentration was higher in septic patients as compared to non-septic patients (7.5 (1.5–41) ng/ml vs. 1.0 (0.3–3.1) ng/ml (*p* < 0.001), respectively). In septic patients, primary reason for mechanical ventilation was acute respiratory failure (38/51, 75%) related to community acquired pneumonia (37/51; 73%). In non-septic patients, main reasons for ventilation were acute respiratory failure not related to community acquired pneumonia (16/41; 39%), coma (13/41; 32%) and cardiac arrest (6/41; 15%). Diaphragm dysfunction was diagnosed in 70% (64/92) of all the patients at inclusion with a median Ptr,stim of 7.6 (5.1–11.8) cmH_2_O. Ptr,stim was lower in septic patients as compared to non-septic patients: 6.3 (4.9–8.7) cmH_2_O vs. 9.8 (7.0–14.2) cmH_2_O (*p* = 0.004), respectively (Fig. 1). Among septic patients, 43/51 (84%) had a diaphragm dysfunction, whereas 21/41 (51%) non-septic patients were diagnosed with diaphragm dysfunction (*p* < 0.001).

**Table 1:** Patients characteristics at inclusion

	Patients with sepsis *n* = 51	Patients without sepsis *n* = 41	*p*
Age, years	64 (53–73)	59 (52–70)	0.323
Male sex, *n* (%)	31 (61)	38 (62)	0.985
Body mass index, kg m^−2^	24 (21–28)	24 (22–26)	0.998
SAPS2	58 (46–70)	52 (36–64)	0.292
SOFA	10 (6–12)	9 (6–11)	0.351
Comorbidities, *n *(%)			
Active smoking	23 (45)	22 (54)	0.414
COPD	10 (20)	11 (27)	0.412
Diabetes mellitus	15 (29)	10 (24)	0.591
Cirrhosis	11 (22)	6 (15)	0.394
Primary reason for ventilation			
Acute respiratory failure, *n* (%)	38 (75)	16 (39)	< 0.001
Pneumonia	37 (73)	0 (0)	< 0.001
Hypercapnic ACRF	0 (0)	6 (15)	0.006
Acute lung edema	0 (0)	2 (5)	0.196
Other	1 (2)	8 (20)	0.009
Shock, *n* (%)	7 (14)	6 (15)	0.985
Septic shock	7 (14)	0 (0)	0.016
Cardiogenic shock	0 (0)	2 (5)	0.110
Other	0 (0)	4 (10)	0.022
Cardiac arrest, *n* (%)	1 (2)	6 (15)	0.023
Coma, *n* (%)	5 (10)	13 (32)	0.008
Stroke	1 (2)	5 (12)	0.048
Hepatic encephalopathy	2 (4)	5 (12)	0.136
Other	2 (4)	3 (7)	0.475
Clinical variables			
Temperature > 38° or < 36°	23 (45)	18 (44)	0.909
Mean arterial pressure, mmHg	78 (70–94)	77 (71–86)	0.780
Ptr,stim, cmH_2_O	6.3 (4.9–8.7)	9.8 (7–14.2)	0.004
Diaphragm dysfunction, *n* (%)	43 (84)	21 (51)	< 0.001
End-expiratory diaphragm thickness, mm	2.3 (1.8–2.7)^a^	2.1 (1.8–2.5)^b^	0.629
Biological variables			
White blood cells count, 10^−9^/l	12.3 (7.5–17.7)	11.6 (10–15.4)	0.845
PCT, ng/ml	7.5 (1.5–41)	1 (0.3–3.1)	< 0.001
Blood lactate, mmol l^−1^	2 (1.2–3)	2 (1.6–3)	0.258
PaO_2_/FiO_2_	201 (144–300)	248 (213–313)	0.013
Sedation			
Hypnotics (propofol or midazolam), *n* (%)	40 (100)^c^	37 (100) ^d^	1.000
Sufentanyl, *n* (%)	35 (88) ^c^	32 (86) ^d^	1.000
Organ support			
Vasopressors, *n* (%)	40 (78)	26 (63)	0.112

Categorical variables are expressed as absolute value (%) and continuous variables as median (interquartile range)

SOFA: Sepsis-related Organ Failure Assessment; SAPS 2: New Simplified Acute Physiology Score; ACRF: acute-on-chronic respiratory failure; Ptr,stim: endotracheal tube pressure induced by bilateral phrenic nerve stimulation during airway occlusion; PCT: procalcitonin; PaO_2_/FiO_2_: partial arterial oxygen tension on inspired oxygen fraction ratio

^a^ Data available for 31/51 patients, ^b^ data available for 27/41 patients

^c^ Data available for 40/51 patients, ^d^ data available for 37/41 patients

Ultrasound measurements were available in 58/92 (63%) of the patients. The diaphragm end-expiratory thickness was not different between septic and non-septic patients upon inclusion (Table 1).

### Time course of diaphragm function and diaphragm thickness

Duration of mechanical ventilation between first and second diaphragm function measurement was 4 (2–6) days in septic patients and 3 (2–4) days in non-septic patients (*p* = 0.073). Between the two measurements, the SOFA score and blood lactate level decreased in septic and non-septic patients (Fig. 1 and see Additional file 1: Table S2). The change in Ptr,stim was + 19% (− 13–61) in septic patients and − 7% (− 40–12) in non-septic patients (*p* = 0.005). In septic patients, Ptr,stim increased from 6.3 (4.8–8.7) cmH_2_O to 7.9 (6.6–11.2) cmH_2_O, whereas it decreased from 9.8 (7.0–14.2) cmH_2_O to 7.3 (4.5–12.8) cmH_2_O in non-septic patients. Between the two measurements, diaphragm function increased in 28 (65%) among the 43 septic patients who had diaphragm dysfunction upon inclusion. In those 28 septic patients, Ptr,stim increased from 5.2 (3.9–6.5) cmH_2_O to 8.6 (6.9–12.1) cmH_2_O. But diaphragm dysfunction was still present at the second measurement in 32/43 of the patients. Only 11/43 patients recovered from the diaphragm dysfunction and had a normal diaphragm function at the second evaluation. In the sub-group of patients with ultrasound measurements, end-expiratory diaphragm thickness decreased in both, septic and non-septic patients (Fig. 1 and see Additional file 1: Table S2).

### Factors associated with an increase in diaphragm function

**Fig. 2:**
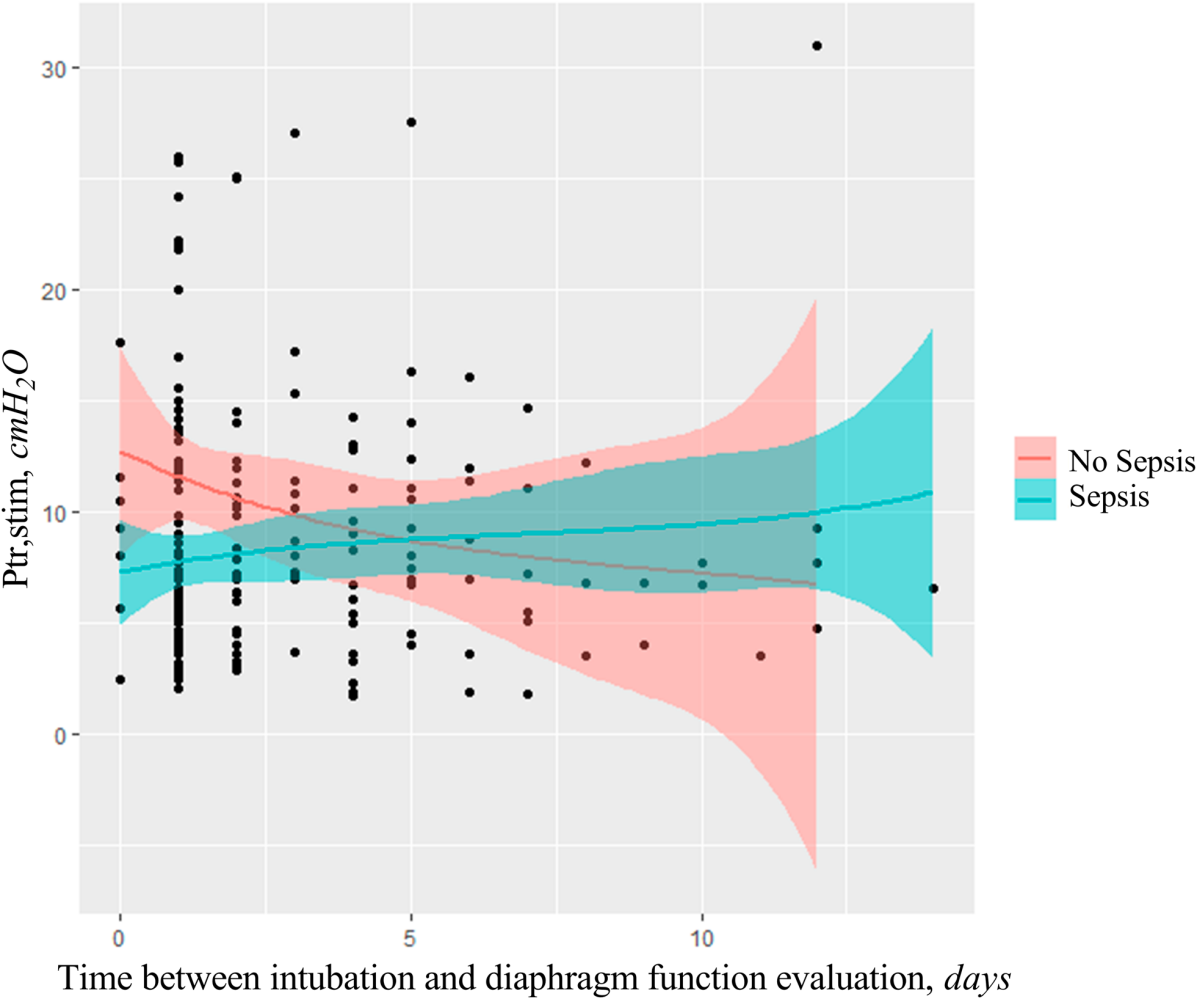
Diaphragm function time course represented by endotracheal tube pressure induced by bilateral phrenic nerve stimulation during airway occlusion (Ptr,stim) according to time between intubation and measure in septic and non-septic patients. Each black circle represents a single measure of the diaphragm function for a given patient (184 circles in total). The lines represent the regression and the colored shaded areas represent 95% confidence interval for the regression curve

Increase in diaphragm function was observed in 40 patients and decrease or no change in diaphragm function was observed in 52 patients (Table 2). In univariate analysis, sepsis at the time of inclusion, body mass index and Ptr,stim at the time of inclusion were associated with an increase in diaphragm function (Table 2). Multivariate analysis found that two factors were independently associated with an increase in diaphragm function, sepsis [Coeff − 4.25 ± 1.24 (SD), *p* < 0.001], and time between intubation and measurement [Coeff − 0.42 ± 0.21 (SD), *p* = 0.046] (see Additional file 1: Table S3). The diaphragm function time course in septic and non-septic patients is depicted in Fig. 2.

**Table 2:** Variables associated with an increase in diaphragm function* and a decrease or no change in diaphragm function between the two measurements

	Increase in diaphragm function *n* = 40	Decrease or no change in diaphragm function *n* = 52	*p*
Time between intubation and second measure, days	5 (3–6)	4 (2–6)	0.163
Time between 2 measures, days	4 (3–5)	3 (1–5)	0.134
Sepsis at inclusion, *n* (%)	29 (73)	22 (42)	0.004
Age, years	61 (49–72)	63 (54–71)	0.425
Male sex, *n* (%)	22 (55)	34 (65)	0.312
Body mass index, kg m^−2^	24 (21–25)	25 (23–28)	0.036
SAPS2 at inclusion	58 (35–66)	54 (45–64)	0.831
SOFA at inclusion	9 (5–11)	10 (7–12)	0.301
Comorbidities, *n* (%)			
Active smoking	20 (50)	25 (48)	0.855
COPD	9 (23)	12 (23)	0.948
Diabetes	11 (28)	14 (27)	0.951
Cirrhosis	7 (18)	10 (19)	0.832
Primary reason for ventilation, *n* (%)			
Acute respiratory failure	28 (70)	27 (52)	0.080
Shock	5 (13)	7 (13)	0.892
Cardiac arrest	1 (3)	6 (12)	0.105
Coma	6 (15)	12 (23)	0.333
Biological variables			
PCT at inclusion, ng/ml	3 (0.3–20)	1.7 (0.9–7.3)	0.909
Blood lactate at inclusion, mmol.l^−1^	1.8 (1.2–3.2)	2.1 (1.4–2.9)	0.838
PaO_2_/FiO_2_ at inclusion	230 (160–305)	226 (162–306)	0.940
PaO_2_/FiO_2_ at second measure	280 (202–327)	260 (220–333)	0.990
Sedation			
Hypnotics at inclusion (propofol or midazolam), *n* (%)	33 (100) ^a^	44 (100) ^c^	1.000
Sufentanyl at inclusion, *n* (%)	29 (88) ^a^	38 (86) ^c^	1.000
Hypnotics at second measure (propofol or midazolam), *n* (%)	19 (56) ^b^	16 (40) ^d^	0.130
Sufentanyl at second measure, *n* (%)	9 (26) ^b^	14 (35) ^d^	0.808
Organ support			
Vasopressors, *n* (%)	28 (70)	38 (73)	0.745
Diaphragm function			
Ptr,stim at inclusion, cmH_2_O	5.8 (4.0–8.0)	10.2 (7.0–14.3)	< 0.001
Ptr,stim at second measure, cmH_2_O	10.5 (7.0–12.5)	6.9 (4.5–9.4)	0.002
Absolute change in Ptr,stim, cmH_2_O	3.4 (1.8–4.8)	– 1.5 (– 4.9 to – 0.6)	< 0.001
Relative change in Ptr,stim, %	59 (32–78)	– 19 (– 43 to – 7)	< 0.001
End-expiratory diaphragm thickness at inclusion, mm	2.3 (1.9–2.9) ^e^	2.1 (1.7–2.5) ^f^	0.484
End-expiratory diaphragm thickness at second measure, mm	1.9 (1.5–2.3) ^e^	2.0 (1.7–2.2) ^f^	0.565

Categorical variables are expressed as absolute value (%) and continuous variables as median (interquartile range)

* Increase in diaphragm function is defined as an increase > 10% of the change of endotracheal tube pressure induced by bilateral phrenic nerve stimulation during airway occlusion (Ptr,stim) between first measure at inclusion and second measure after switch to pressure support mode

SOFA: Sepsis-related Organ Failure Assessment; SAPS 2: New Simplified Acute Physiology Score; COPD: chronic obstructive pulmonary disease; PCT: procalcitonin; PaO_2_/FiO_2_: partial arterial oxygen tension on inspired oxygen fraction ratio; Ptr,stim: endotracheal tube pressure induced by bilateral phrenic nerve stimulation during airway occlusion

^a^ Data available for 33/40 patients, ^b^ data available for 34/40 patients

^c^ Data available for 44/52 patients, ^d^ data available for 40/52 patients

^e^ Data available for 28/40 patients

^f^ Data available for 30/52 patients

### Clinical outcomes

Overall, ICU mortality was 28% (26/92). Ventilatory free days at 28 days, total length of ICU stay, ICU and 28 days mortality were not different between septic and non-septic patients (see Additional file 1: Table S4). Mortality at day 28 was higher in patients with decrease or no change in diaphragm function than in patients with an increase in diaphragm function (Table 3).

**Table 3:** Main outcomes according to the time course of diaphragm function

	Increase in diaphragm function* *n* = 40	Decrease or no change in diaphragm function *n* = 52	*p*
Total duration of mechanical ventilation, days	7 (5–15)	7 (5–14)	0.593
Ventilatory free days at 28 days, days	18 (2–23)	15 (0–23)	0.399
Total length of ICU stay, days	13 (7–21)	10 (7–19)	0.687
ICU mortality, *n* (%)	8 (20)	18 (35)	0.123
Mortality at day 28, *n* (%)	9 (23)	22 (42)	0.046

Categorical variables are expressed as absolute value (%) and continuous variables as median (interquartile range)

ICU: intensive care unit

^*^ Increase in diaphragm function is defined as an increase > 10% of the change of endotracheal tube pressure induced by bilateral phrenic nerve stimulation during airway occlusion between first measure at inclusion and second measure performed at the time of first switch to pressure support mode

## Discussion

In this series of patients, we investigated the effect of sepsis in combination with invasive mechanical ventilation on the diaphragm function time course of critically ill patients admitted to the ICU for various reasons. This study reports an association between the presence of sepsis at the time of inclusion and the reversibility of the decrease in diaphragm pressure generating capacity in critically ill patients despite the exposure to mechanical ventilation. Our study therefore provides evidences supporting the hypothesis of reversible sepsis-associated impaired diaphragm function.

It is well established that mechanical ventilation induces a time-dependent diaphragm dysfunction in patients admitted in the ICU [23, 24]. However, beyond the negative impact of time-dependent mechanical ventilation-induced respiratory muscles unloading on diaphragm function, other contributors of “ICU-induced diaphragm dysfunction” have been reported [6]. Among them, sepsis is likely to play a leading role [25, 26]. Sepsis impairs diaphragm force apart from any effect on muscle mass or architecture [27], which suggests that systemic inflammation is an important determinant in this context. Sepsis may act at two levels on the occurrence of diaphragm dysfunction [6]. First, it can alter the chain of muscular energy supply through impairment in blood flow distribution (hypoxic ischemia) and use (cytopathic ischemia). Second, it can be responsible of a dysfunction of the contractile proteins induced by cytokines, in particular the tumor necrosis factor alpha (TNF-α) [28]. The potential interaction between sepsis and mechanical ventilation is not univocal. On one hand, mechanical ventilation-induced respiratory muscles unloading may prevent the diaphragm to contract in a septic environment [29]. On the other hand, mechanical ventilation-induced respiratory muscles unloading can lead to diaphragm atrophy [30] and a time-dependent dysfunction [23, 24]. Same pathogenetic mechanisms, such as increased oxidative stress and mitochondrial dysfunction have been consistently reported in animal models of sepsis-associated diaphragm dysfunction and ventilator-induced diaphragm dysfunction [31]. Therefore, it has been suggested that mechanical ventilation may play as a second hit in combination with sepsis and could create a ‘perfect storm’, with mechanical ventilation either exacerbating the magnitude of diaphragm dysfunction caused by infection or slowing the subsequent recovery of diaphragm function once sepsis has resolved [32]. Our study brings novel insights in this context by providing new evidences of reversible sepsis-associated diaphragm dysfunction. Given the high mortality with sepsis, this may have constituted a selection bias and influenced our results which therefore warrant confirmation. Our results suggest that the effects of sepsis and mechanical ventilation on diaphragm function are not synergistic and that sepsis may induce a reversible decrease in diaphragm pressure generating capacity. Our study confirms animal models [8, 33] by showing a more pronounced diaphragm dysfunction in septic patients as compared to non-septic patients. Interestingly, while the disease severity of both groups improved between two measurements (the SOFA score decreases in both groups), the non-septic group was associated with a decrease in diaphragm function (− 7%), whereas the septic group was associated with an increase in diaphragm function (+ 19%). The definition of sepsis used in our study is of course debatable and uncontrolled factors may certainly have affected the diaphragm function (lung volume [34], systemic inflammation [22], sedatives [35]) and influenced our results. Nevertheless, relevant differences between septic and non-septic groups can be noted regarding the characteristics of the patients (more hypoxemic acute respiratory failure in septic patients, more coma in non-septic patients) and the plasma procalcitonin concentration (significantly higher in septic patients) which soundly suggest that an infection was present in patients classified as being sepsis. In addition, it is somehow reassuring that microbiological findings were found in 40/51 (78%) of the septic patients which was not the case of the non-septic patients.

While we did not evaluate the cardiac function in our patients, our findings echo the already described phenomenon of sepsis-induced myocardial dysfunction [36]. The diaphragm and the heart are both striated skeletal muscles that are susceptible to sepsis. The seminal observation of sepsis-related myocardial dysfunction reported a gradual return to normal ejection fraction and ventricular volume by 10 days after the onset of shock in survivors [16]. Whether the reversible sepsis-associated diaphragm dysfunction time course follows or not the same evolution than sepsis-related myocardial dysfunction remains to be elucidated in further studies. In addition, whether the two diseases coexist or not have never been reported so far and should be further investigated. Notwithstanding, the pathophysiological mechanisms leading to sepsis-associated diaphragm dysfunction and sepsis-induced myocardial dysfunction are different [26, 31, 37]. A major difference being that by contrast to the respiratory muscles, the myocardial muscle is not subject to forced rest.

In our study, a decreased in diaphragm thickness was observed independently of the septic status of the patients [38] while a more important decrease in diaphragm thickness would have been expected in the septic patients because of inflammation-mediated mechanisms [9]. For instance, Jung et al. found that both psoas and diaphragm volumes decreased in 23 critically ill patients with a predominant decrease among the 14 septic patients [9]. However, the former study used computed tomography that offers a 3 dimensions evaluation of the muscle mass, whereas ultrasound might be limited since it usually provides a 2 dimensions estimate. Notwithstanding, time spent under mechanical ventilation is a well established risk factor of diaphragm atrophy [39] and it is very possible that diaphragm atrophy occurred in septic and non-septic patients. The lack of difference in diaphragm thickness changes between septic and non-septic patients could also be ascribed to the limited accuracy of diaphragm ultrasound to detect small changes in diaphragm thickness and to the relatively small number of patients in whom diaphragm ultrasound data were available [40]. Larger studies will have to confirm these findings. The measurement of diaphragm stiffness by shear wave elastography [41–43], a recent ultrasound technique, enables to characterize the structure of the diaphragm may be useful to further address this issue.

A striking result of our study is the association between the increase in diaphragm pressure generating capacity and a better day-28 survival. It has been suggested that sepsis-associated diaphragm dysfunction may behave as any other sepsis-associated organ failure [5], therefore an increase in diaphragm function over the ICU stay is consistent with the decrease in SOFA score and the observed better 28-day survival.

### Strengths and weaknesses

To the best of our knowledge, our study is the first to analyze the impact of the sepsis on the diaphragm function time course under mechanical ventilation. It is constituted of large sample of critically ill patients intubated for various reasons admitted in two ICUs.

Our study has limitations. First, our cohort has only intubated patients which does not allow to study the effects of sepsis on the diaphragm of non-ventilated patients. Second, our dataset does not provide any data regarding the cytokines profiles of our patients. Further studies will have to investigate the parallel time course of the diaphragm function and inflammatory mediators. Third, the level of inspiratory effort was not collected which precludes evaluating the effect of maintaining a diaphragm contractile activity in a septic environment. Fourth, the timing between the two measurements was not standardized. As every patient is characterized by a distinct evolution course, we opted to match the design of the study along individual patient evolutions. The time spent under mechanical ventilation could be an important confounder when comparing septic and non-septic patients, but the multivariate analysis provided reassuring conclusion on this important point. Fifth, the diaphragm function was only assessed at two time points and the evolution beyond the second measurement still remains unknown. Notably, the diaphragm function time course of septic patients who deceased before the second the evaluation is not reported here. For obvious ethical reasons, diaphragm function assessment measurement was not repeated in patients with worsening condition. Finally, this study was not powered to assess clinical outcomes like mortality so the better day-28 survival for patients with increase in diaphragm pressure generating capacity will need to be confirmed by further studies.

## Conclusion

As compared to non-septic patients, septic patients were associated with a severe diaphragm dysfunction that improved over the ICU stay despite the exposure to mechanical ventilation. An increase in diaphragm function was associated with a better survival.

## Supplementary Information

**Additional file 1.** This file contains four more tables with additional results and also the flow chart of the study.

## Data Availability

M. Lecronier is the guarantor of the content of the manuscript, including the data and analysis. The datasets analyzed during the current study are available from the corresponding author on reasonable request.

## Abbreviations

ICU: Intensive care unit,
PEEP: Positive end-expiratory pressure,
SOFA: Sequential Organ Failure Assessment,
SAPS 2: New Simplified Acute Physiology Score
