# Supplementary material for: Severe but reversible impaired diaphragm function in septic mechanically ventilated patients
Source: Ann Intensive Care. 2022 Apr 11;12:34. doi: 10.1186/s13613-022-01005-9 (PMC9001790; doi:10.1186/s13613-022-01005-9)
Supplement: Supplementary file 1 — Additional file 1. This file contains four more tables with additional results and also the flow chart of the study. [file 13613_2022_1005_MOESM1_ESM.docx]

**Supplemental Digital Content**

**Supplementary tables**

**Table 1.** Clinical and microbiological findings in patients with sepsis

|  | **Patients with sepsis**  **n = 51** |
| --- | --- |
| **Source of infection*** |  |
| Respiratory tract infection | 41 (80) |
| Digestive tract infection | 6 (12) |
| Urinary tract infection | 2 (4) |
| Cutaneous infection | 2 (4) |
| Bloodstream infection | 11 (22) |
|  |  |
| **Microbiological findings*** | 40 (78) |
| Gram negative bacilli | 18 (45) |
| *Enterobacteriaceae* | 10 (25) |
| *Echerichia coli* | 4 (10) |
| *Klebsiella pneumoniae* | 3 (8) |
| *Enterobacter cloacae* | 4 (10) |
| *Haemophilus influenzae* | 5 (13) |
| *Pseudomonas aeruginosa* | 6 (15) |
| *Stenotrophomonas maltophilia* | 1 (3) |
| Gram positive cocci | 18 (45) |
| *Staphylococcus aureus* | 5 (13) |
| Coagulase-negative staphylococci | 2 (5) |
| *Enterococcus sp.* | 4 (10) |
| *Streptococcus sp.* | 9 (23) |
| *Streptococcus pneumoniae* | 7 (18) |
| *Pneumocystis jiroveci* | 5 (13) |
| *Influenzae virus* | 2 (5) |
| *Candida* sp. | 2 (5) |
| Polymicrobial infection | 15 (38) |

Categorical variables are expressed as absolute value (%).

*****A given patient could have several sources of infection and polymicrobial infection, which explains that the sum could be greater than 100%.

**Table 2.** Changes in patients’ characteristics between the two measurements.

|  | **Patients**  **with sepsis (n=51)** | | |  | **Patients**  **without sepsis (n=41)** | | |
| --- | --- | --- | --- | --- | --- | --- | --- |
|  | Inclusion | Pressure support | p |  | Inclusion | Pressure support | p |
| Duration of MV since intubation, *n (%)* | 1 (1-1) | 5 (3-7) | <0.001 |  | 1 (1-1) | 4 (3-5) | <0.001 |
| Duration of MV since inclusion, *n (%)* | - | 4 (2-6) | - |  | - | 3 (2-4) | - |
| SOFA score | 10 (6-12) | 6 (4-8) | <0.001 |  | 9 (6-11) | 7 (4-10) | 0.033 |
| **Ventilator parameters** |  |  |  |  |  |  |  |
| Tidal volume, *ml/kg IBW* | 6 (6-7) | 7 (6-9) | 0.017 |  | 6 (5-7) | 6 (5-8) | 0.088 |
| PEEP, *cmH_2_O* | 6 (5-10) | 6 (5-8) | 0.551 |  | 5 (5-6) | 5 (5-6) | 0.149 |
| Respiratory rate, *min^-1^* | 22 (18-28) | 24 (19-25) | 0.508 |  | 21 (17-24) | 20 (16-24) | 0.439 |
| **Clinical variables** |  |  |  |  |  |  |  |
| Mean arterial pressure, *mmHg* | 78 (70-94) | 78 (70-94) | 0.390 |  | 77 (71-86) | 81 (73-90) | 0.355 |
| Heart rate, *min^-1^* | 100 (88-110) | 90 (84-108) | 0.989 |  | 86 (69-96) | 91 (78-103) | 0.473 |
| **Sedation** |  |  |  |  |  |  |  |
| Hypnotics (Propofol or Midazolam), *n (%)* | 40 (100)^a^ | 18 (47) ^b^ | <0.001 |  | 37 (100) ^c^ | 17 (47) ^d^ | <0.001 |
| Sufentanyl, *n (%)* | 35 (88) ^a^ | 12 (32) ^b^ | <0.001 |  | 32 (86) ^c^ | 11 (31) ^d^ | <0.001 |
| **Arterial blood gases** |  |  |  |  |  |  |  |
| Blood lactate, *mmol.l^-1^* | 1.9 (1.2-2.9) | 1.5 (1.1-1.9) | 0.035 |  | 2.2 (1.5-3.0) | 1.5 (1.1-1.9) | <0.001 |
| pH | 7.37 (7.28-7.44) | 7.43 (7.40-7.45) | <0.001 |  | 7.40 (7.32-7.45) | 7.43 (7.36-7.45) | 0.059 |
| PaCO_2_, *mmHg* | 37 (33-45) | 38 (33-47) | 0.282 |  | 37 (32-43) | 39 (35-48) | 0.062 |
| PaO_2_/FiO_2_ | 201 (144-300) | 280 (208-318) | 0.014 |  | 248 (213-313) | 266 (223-340) | 0.753 |
| **Diaphragm assessment** |  |  |  |  |  |  |  |
| Ptr,stim, *cmH_2_O* | 6.3 (4.9-8.7) | 7.9 (6.7-11.2) | 0.054 |  | 9.8 (7.0-14.2) | 7.3 (4.5-12.8) | 0.084 |
| Diaphragm dysfunction, *n (%)* | 43 (84) | 37 (73) | 0.149 |  | 21 (51) | 27 (66) | 0.179 |
| End expiratory diaphragm thickness, *mm* | 2.3 (1.8-2.7)^e^ | 2.0 (1.6-2.3) ^e^ | <0.001 |  | 2.1 (1.8-2.5)^f^ | 1.9 (1.6-2.2) ^f^ | 0.002 |

Categorical variables are expressed as absolute value (%) and continuous variables as median (interquartile range).

MV: Mechanical ventilation; IBW: Ideal body weight; FiO_2_, Inspired oxygen fraction; PaO_2_/FiO_2_, Partial arterial oxygen tension on inspired oxygen fraction ratio;

PEEP: Positive end expiratory pressure; Ptr,stim, Endotracheal tube pressure induced by bilateral phrenic nerve stimulation during airway occlusion.

^a^ Data available for 40/51 patients, ^b^ Data available for 38/51 patients

^c^ Data available for 37/41 patients, ^d^ Data available for 36/41 patients

^e^ Data available for 31/51 patients

^f^ Data available for 27/41 patients

**Table 3.** Univariate and multivariate analysis of the variables associated with an increase in diaphragm function.

| **Variables** | **Event** | | **Univariate analysis** | |  | **Mixed Linear Model** | | | |
| --- | --- | --- | --- | --- | --- | --- | --- | --- | --- |
|  |  | |  | **P value** |  | **Coefficient** | **SD** |  | **P value** |
| Time between intubation and measure, *days* | Increase in diaphragm function | | 5 (3-6) | 0.163 |  | –0.42 | 0.21 |  | 0.046 |
|  | Decrease or no change in diaphragm function | | 4 (2-6) |  |  |  |  |  |  |
| Sepsis at inclusion, *%* | Increase in diaphragm function | | 73 | 0.004 |  | –4.25 | 1.24 |  | <0.001 |
|  | Decrease or no change in diaphragm function | | 42 |  |  |  |  |  |  |
| Interaction between Time between intubation and  measure and Sepsis at inclusion | |  | NA | NA |  | 0.72 | 0.25 |  | 0.006 |

**Table 4.** Main outcomes according to the sepsis status.

|  | **All**  **patients**  **n=92** | **Patients**  **with sepsis**  **n=51** | **Patients**  **without sepsis**  **n=41** | **p** |
| --- | --- | --- | --- | --- |
| Total duration of mechanical ventilation | 7 (5-15) | 8 (5-15) | 5 (5-10) | 0.201 |
| Ventilatory free days at 28 days | 17 (0-23) | 16 (1-23) | 18 (0-23) | 0.659 |
| Total length of ICU stay | 11 (7-20) | 13 (8-21) | 8 (7-18) | 0.138 |
| ICU mortality, *n (%)* | 26 (28) | 12 (24) | 14 (34) | 0.261 |
| Mortality at day 28, *n (%)* | 31 (34) | 14 (27) | 17 (41) | 0.158 |

Categorical variables are expressed as absolute value (%) and continuous variables as median (interquartile range).

ICU: Intensive care unit.

**Legend of the supplementary figures**

**Figure 1.** Flow chart of the study.

**Figure 1.**
